# Biomonitoring Studies Should Be Used by Regulatory Agencies to Assess Human Exposure Levels and Safety of Bisphenol A

**DOI:** 10.1289/ehp.0901717

**Published:** 2010-05-05

**Authors:** Laura N. Vandenberg, Ibrahim Chahoud, Vasantha Padmanabhan, Francisco J.R. Paumgartten, Gilbert Schoenfelder

**Affiliations:** 1 Tufts Center for Regenerative and Developmental Biology and; 2 Department of Biology, Tufts University, Medford, Massachusetts, USA; 3 Institut für Klinische Pharmakologie und Toxikologie, Charité Universitätsmedizin Berlin, Campus Benjamin Franklin, Berlin, Germany; 4 Department of Pediatrics; 5 Department of Obstetrics and Gynecology and; 6 Department of Molecular and Integrative Physiology, University of Michigan, Ann Arbor, Michigan, USA; 7 Laboratory of Environmental Toxicology, National School of Public Health, Oswaldo Cruz Foundation, Rio de Janeiro, Rio de Janeiro, Brazil; 8 Institute of Pharmacology and Toxicology, University of Würzburg, Würzburg, Germany

**Keywords:** bisphenol A, endocrine disruptor, Good Laboratory Practices, human exposure, regulatory policy, risk assessment, toxicokinetic model

## Abstract

**Background:**

Within the past 3 years, four major evaluations of bisphenol A (BPA) safety have been undertaken. However, these assessments have arrived at quite different conclusions regarding the safety of BPA at current human exposure levels.

**Objectives:**

We compared the reasons provided by the European Food Safety Authority (EFSA) BPA risk assessment panel for their conclusion that human exposures are negligible with the conclusions reached by the other panels, with all panels having the same body of literature at their disposal.

**Discussion:**

The EFSA panel dismissed ≥ 80 biomonitoring studies that documented significant levels of BPA exposure in humans, including internal exposures to unconjugated BPA, on the basis that they did not match a model of BPA metabolism. Instead, the EFSA panel relied on two toxicokinetic studies—conducted in 15 adults administered BPA—to draw conclusions about exposure levels in the population, including exposures of neonates.

**Conclusions:**

As with all exposure assessments, models should be developed to explain actual data that are collected. In the case of BPA, samples from a large number of human subjects clearly indicate that humans are internally exposed to unconjugated BPA. The dismissal of these biomonitoring studies simply because their results do not conform to a model violates scientific principles. Expert panels should evaluate all data—including human biomonitoring studies—to make informed risk assessments.

Bisphenol A (BPA), a component of polycarbonate plastics and epoxy resins, is one of the highest-volume chemicals produced worldwide. Many studies suggest that the amount of BPA to which humans are exposed may cause adverse health effects (reviewed by Bondesson et al. 2009).

The field of endocrine disruption, and particularly BPA research, has been influenced by social issues, legislation, and the media. BPA has attracted the attention of regulatory agencies and scientists around the world because of its estrogenic properties (Wetherill et al. 2007). Since 2006, several panels and agencies have examined the BPA literature and have come to quite different conclusions regarding the safety of human exposure levels [reviewed by Gies et al. (2009) and Vandenberg et al. (2009)]. Specifically, exposure of humans to free (unconjugated) BPA has been questioned. These conflicting decisions seem paradoxical because each was generated using approximately the same literature database.

## EFSA Risk Assessment: An Example of Use of Limited Data

As stated in our review (Vandenberg et al. 2010), great concern exists about exposure of human fetuses, infants, and neonates to BPA because of the sensitivity of developing organs and the brain to exogenous hormones (Vandenberg et al. 2009). However, to translate findings from animal studies to health risks in humans, exposure assessments and biomonitoring of BPA in different populations are essential. Thus, in November 2006, the European Food Safety Authority (EFSA) released its opinion on the plausibility of data regarding levels of BPA in human blood and excretion of BPA and BPA metabolites in environmentally exposed humans. The EFSA panel (2006) concluded that

[T]here is very low oral bioavailability of the parent substance, BPA, in humans and other primates. Due to this rapid biotransformation and excretion and plasma protein binding in humans, peak BPA-concentrations after dietary exposures to BPA available for receptor binding are predicted to be very low even in worst case exposure scenarios.

The EFSA panel was asked to reconsider their assessment based on recent studies that suggested the possibility for age-dependent toxicokinetics of BPA. In July 2008, the EFSA released its second opinion in support of their original statement (EFSA 2008):

The Panel therefore considers that its previous risk assessment … can be considered as conservative for humans. The Panel concluded that the differences in age-dependent toxicokinetics of BPA in animals and humans would have no implication for the EFSA 2006 risk assessment of BPA.

In stark contrast to these statements, we analyzed > 80 biomonitoring studies and came to the conclusion that measurable levels of BPA and BPA conjugates are present in human blood and urine, as well as in other tissues and fluids (Vandenberg et al. 2010). These biomonitoring studies examined thousands of individuals from many developed and some developing countries and collectively indicate that humans are internally exposed to unconjugated BPA (Vandenberg et al. 2007, 2010; Welshons et al. 2006). Biomonitoring studies are crucial for understanding current human exposure levels because, by their very nature, they account for all exposures. This is essential, because all exposure sources for BPA have not yet been identified, and existing data suggest that non-oral exposures may be significant (Gies et al. 2009; Stahlhut et al. 2009).

A comprehensive review of the large number of biomonitoring studies indicates that they are highly consistent and therefore reliable (Vandenberg et al. 2010). The detection rates and concentrations of BPA in urine and blood of environmentally exposed individuals are remarkably similar in studies performed in many laboratories using a variety of techniques, including highly accurate and sensitive methods [e.g., solid-phase extraction coupled with isotope dilution-HPLC-tandem mass spectrometry, as used by the U.S. Centers for Disease Control and Prevention (Calafat et al. 2005, 2008)]. Further, there is no evidence to suggest that these studies should be invalidated because of poor quality control (e.g., contamination from collection materials, breakdown of conjugates during storage, inadequate blanks) (Gies et al. 2009; Vandenberg et al. 2010). In total, the reproducibility of these results indicates that humans are internally exposed to doses of unconjugated BPA, with a central measure of the distribution in the 0.5–3 ng/mL range. In spite of these consistent findings, the EFSA panel came to a completely different conclusion about current human exposures (EFSA 2006, 2008). What is the basis for this disparity?

The pivotal factor we identified in the EFSA report was the bias in the selection of studies used in this evaluation. The EFSA panel (EFSA 2006) ignored the majority of the biomonitoring studies. Although they reviewed 2 toxicokinetic studies (Volkel et al. 2002, 2005) extensively, only 2 of the 17 urine biomonitoring studies published by 2006 were discussed in any detail. Only a small number of the blood biomonitoring studies were cited in the EFSA report, and none of these studies were discussed in detail at any level. Instead, the EFSA panel identified potential problems with these biomonitoring studies, including the use of ELISA (used only in a few studies), possible contamination of reagents with BPA, and the leaching of BPA from materials used for sample collection, storage, and processing. Without providing any evidence that these are indeed issues in the biomonitoring studies examined, the EFSA (2006) concluded that

Due to all these confounders, the reported analytical results on BPA blood concentrations most probably considerably overestimate real blood concentrations actually present.

Of particular concern relative to this stance is that although the biomonitoring studies have produced reliable, consistent results, the two toxicokinetic studies (Volkel et al. 2002, 2005) the EFSA (2006) relied upon heavily for their risk assessment have significant inconsistencies and are yet to be replicated. Most concerning is the fact that the methods used in these two toxicokinetic studies were much less sensitive than those used in almost all biomonitoring studies. The toxicokinetic studies had limits of detection (LODs) as high as 2.28 ng/mL for unconjugated BPA and 10.1 ng/mL for conjugated BPA, compared with LODs of 0.0063–0.4 ng/mL in other studies using similar analytical methods (Vandenberg et al. 2010). These two toxicokinetic studies examined only a small number of adult subjects administered BPA (15 adults total) compared with the thousands of individuals (including infants, children, adolescents, and pregnant women) sampled for biomonitoring purposes. Only one of these studies (Volkel et al. 2002) examined concentrations of BPA in both blood and urine samples, whereas the other study (Volkel et al. 2005) reported conjugated BPA concentrations in urine but provided no information about BPA concentrations in the plasma samples collected by the authors. Yet both studies were used by the EFSA to discount the presence of BPA in plasma and blood samples reported in numerous other studies (EFSA 2006). Additional problems with data analysis and interpretation in the [Bibr b30-ehp.0901717][Bibr b29-ehp.0901717]) are discussed in greater detail in our review (Vandenberg et al. 2010).

The EFSA panel (2006) speculated that the repeated detection of unconjugated BPA in human blood was due to poor sample processing conditions and/or unreliable methods, stating,

The studies reporting detection of BPA in human blood in concentrations higher than 1 [μg] BPA/L have usually determined [unconjugated] BPA, without prior enzymatic cleavage of BPA-glucuronide.… The fate of BPA-glucuronide under the conditions of the diverse sample processing conditions and a possible cross-reactivity of the [ELISA] antibodies with BPA-glucuronide is not reported, leaving the possibility that reported BPA levels actually reflect BPA-glucuronide levels.

Consistent results from a large number of biomonitoring studies cannot be disregarded based only on the speculation that they overestimated unconjugated BPA levels because of hypothetical poor analytical controls. The deficiencies speculated by the EFSA were addressed and invalidated by one or more appropriate controls within each of the individual biomonitoring studies in question; most studies contained numerous controls to counter speculations of contamination or cross-reactivity of ELISA antibodies. For example, blanks reported in these studies would show measurable BPA if cross-contamination occurred at any step in the sample-handling process or analysis—yet they did not, leaving the speculations made by the EFSA without any scientific basis.

The EFSA panel (EFSA 2006) continued to rationalize their dismissal or lack of attention to biomonitoring studies by referencing the results of toxicokinetic studies:

[O]rally administered BPA is rapidly absorbed from the gastrointestinal tract and undergoes intensive first-pass metabolism to BPA-glucuronide in the gut wall and in the liver.… Concentrations of [unconjugated] BPA were below the limit of detection both in urine … and blood samples….

Further reasoning provided to reject the findings from biomonitoring studies was that the levels measured in environmentally exposed humans are “higher than the peak BPA concentrations determined in blood of monkeys after oral administration of a dose of 100 μg BPA/kg bw [body weight].” The panel concluded that

[T]hese reported concentrations of BPA in blood of unintentionally exposed human subjects of up to 10 [μg] BPA/L are orders of magnitude above the maximal concentrations of BPA predicted in blood by PBPK [physiologically-based pharmacokinetic] models on the basis of human BPA toxicokinetics after oral administration.

In science, if data contradict the hypothesis (i.e., the model), the hypothesis, not the data, must be rejected. It is unexpected, and perhaps unprecedented, for a scientific body to reject studies because their findings did not match a model, rather than to reconsider the model or reassess the findings from the extremely limited toxicokinetic studies that were used to generate the model. This reasoning is simply not founded in logic and is not how science-based regulatory decisions should be made. Considering the size of the biomonitoring literature, the consistency of the results from biomonitoring studies, and the significant problems in the toxicokinetic studies, conclusions drawn primarily from the two toxicokinetic studies (Volkel et al. 2002, 2005) cannot be valid. Therefore the EFSA conclusion that there is negligible internal exposure to unconjugated BPA has no scientific basis.

## The EFSA Panel Inappropriately Extrapolates from Adults to Fetuses and Neonates

Considering the reliance of the EFSA panel on two extremely limited toxicokinetic studies to inform their risk assessment, their statement that “the differences in age-dependent toxicokinetics of BPA in animals and humans would have no implication for the EFSA 2006 risk assessment of BPA” (EFSA 2008) is particularly surprising. The July 2008 EFSA report stated,

The Panel considers that there is sufficient capacity in the neonate to conjugate BPA at doses below 1 mg/kg bw (the Panel noted that exposures at the TDI of 0.05 mg/kg bw are 20 fold lower than this). Therefore, the Panel concluded that there is sufficient capacity for biotransformation of BPA to hormonally inactive conjugates in neonatal humans at exposures to BPA that were considered in the EFSA opinion of 2006 and the European Union Risk Assessment Report.

To date, there are no studies to support this statement. To the contrary, there are many studies that contradict it. First, the two toxicokinetic studies relied upon by EFSA (Volkel et al. 2002, 2005) examined a total of 15 adults (mixed groups of males and females) administered BPA. Although the authors of these studies concluded that there are no kinetic differences between volunteers (Volkel et al. 2005), evaluation of the data presented shows variable metabolic responses after BPA administration. Second, data from biomonitoring studies in different groups of adults clearly indicated differences in urinary concentrations of BPA that are influenced by both sex and age (Calafat et al. 2005, 2008; He et al. 2009). Associations between age and BPA concentrations are also evident from studies that examined children and adolescents; younger children typically have higher concentrations of BPA metabolites in urine compared with older children and adolescents (Becker et al. 2009; Calafat et al. 2008). Infants in a neonatal infant care unit were found to have total urinary BPA concentrations approximately 11 times higher than those observed in adults (Calafat et al. 2009). Third, researchers using two physiologically based toxicokinetic models that simulated the blood concentration time profile in several age groups predicted that newborns have 3–11 times greater blood BPA concentrations than adults (Edginton and Ritter 2009; Mielke and Gundert-Remy 2009). Finally, a recently published study examining rat fetuses provides evidence that BPA-glucuronide passes from the mother through the placenta and is deconjugated to BPA in the fetus, clearly showing that BPA metabolites can be converted to the biologically active form in the fetus (Nishikawa et al. 2010). A study of human placentas also indicates that unconjugated BPA crossing the placental barrier remains largely in its unconjugated form. Less than 4% of BPA detected in the fetal compartment was conjugated (Balakrishnan et al. 2010).

Similarly, there is little evidence in support of complete conjugation of BPA, even in adults. Six of the seven biomonitoring studies testing for unconjugated BPA in urine found measurable concentrations in at least some individuals examined (Calafat et al. 2009; Kim et al. 2003; Ouichi and Watanabe 2002; Schoringhumer and Cichna-Markl 2007; Volkel et al. 2008; Ye et al. 2005). The one study that failed to detect unconjugated BPA examined five pooled urine samples (Brock et al. 2001). One of the toxicokinetic studies relied heavily upon by the EFSA (2006, 2008) also detected unconjugated BPA in the urine of two of the six individuals administered BPA (Volkel et al. 2005). The presence of unconjugated BPA in urine suggests that first-pass metabolism of orally administered BPA may be incomplete, that significant levels of BPA enter the body via routes that circumvent first-pass metabolism, or that BPA metabolites are deconjugated in the body. Importantly, unconjugated BPA has also been measured in fetal umbilical cord blood, amniotic fluid, and placental tissue (Vandenberg et al. 2010). Collectively, these findings clearly indicate that the fetus does not have “sufficient capacity for biotransformation of BPA to hormonally inactive conjugates” (EFSA 2008) and that human adults may not either.

## Divergent Conclusions from Other Expert Panels

In the past few years, three other major evaluations of the BPA toxicological database have been undertaken. These expert panels came to seemingly disparate conclusions, yet all four evaluations took place within a short period of time and had access to essentially the same literature. How is it possible for the same studies to be reviewed so differently by regulatory agencies [the EFSA and the U.S. Food and Drug Administration (FDA)], the National Toxicology Program (NTP), and academic scientists?

The answer lies in how the various panels evaluated the scientific literature. In a previous commentary in *Environmental Health Perspectives*, Myers et al. (2009) described the selection process used by each panel in its assessment of the hundreds of animal studies that, to date, overwhelmingly indicate that developmental exposure to BPA causes adverse effects.The EFSA (2006) and FDA (2008) assessments used only data produced using validated protocols [i.e., studies that conformed to Good Laboratory Practices (GLP)] with the ability to establish no observed adverse effect levels. Although the EFSA and FDA stated that they would use all available data to make regulatory decisions, their guidelines restricted their focus to only a few GLP-compliant studies; all other studies (nearly 1,000 for BPA) were not used because they did not meet this criterion.

Similarly, the EFSA panel (EFSA 2006) clearly divided the human exposure database into two groups: dozens of biomonitoring studies (which did not fit their model of BPA metabolism and were largely ignored or rejected) and two toxicokinetic studies (which fit their model and were used in spite of their higher LODs and small number of individuals examined). The studies used by the other three expert panels and the conclusions reached by each of these panels are discussed below.

## Chapel Hill Consensus Statement

In the fall of 2007, a group of scientists from universities and government agencies developed a workshop sponsored by the National Institutes of Health to which experts researching BPA and other endocrine-disrupting chemicals were invited. These academic scientists wrote the Chapel Hill Consensus Statement (vom Saal et al. 2007), which stated, in part, that “the commonly reported circulating levels in humans exceed the circulating levels extrapolated from acute exposure studies in laboratory animals.”

In reaching these conclusions, vom Saal et al. (2007) examined the entire body of scientific data (Crain et al. 2007; Keri et al. 2007; Richter et al. 2007; Vandenberg et al. 2007; Wetherill et al. 2007), including > 40 human biomonitoring studies available at the time and the two human toxicokinetic studies. The panel concluded that humans, including children, adult men and women, and pregnant women, have measurable levels of unconjugated BPA in their bodies, stating succinctly that “[h]uman exposure to BPA is widespread” (vom Saal et al. 2007). Additionally, a subpanel of experts (Vandenberg et al. 2007) concluded that

Unconjugated BPA has been measured repeatedly in human blood (serum and plasma) with a central measure of the distribution in the 0.3–4.4 ng/ml range (1–19.4 nM), and in breast milk, amniotic fluid, and placental tissue in the low [nanograms per milliliter] or [nanograms per gram] range.

## NTP

During the same period of time, the NTP Center for the Evaluation of Risks to Human Reproduction (CERHR) established a committee to evaluate the effects of BPA on reproductive health in humans (CERHR 2007). The original CERHR report, and several subsequent drafts, were challenged and harshly criticized by scientists because they used arbitrary criteria to evaluate animal studies, applied these criteria unevenly to different studies, and contained scientific errors and misinterpretations of published data [reviewed by Vandenberg et al. (2009)]. In the spring of 2008, the NTP undertook its own extensive review of the BPA literature, including recommendations from the CERHR report and comments from the public (NTP 2008).

The NTP (2008) limited its review to those studies related to risks for human reproduction; most of the human exposure studies available at the time were included in the assessment, whereas only a portion of the animal literature was considered useful. Regarding human exposures, the NTP came to a much less decisive conclusion compared with the Chapel Hill panel (vom Saal et al. 2007), stating that “there are data reporting bisphenol A concentrations in urine, breast milk, and amniotic fluid.” Yet, the NTP (2008) also stated that the many biomonitoring studies may be unreliable because BPA conjugates can be unstable under some storage conditions and because laboratory equipment may leach BPA: “it is possible that free bisphenol A concentrations measured in biological samples may be overestimated.” Similar to the EFSA report (EFSA 2006), the NTP (2008) reached these conclusions without evidence that contaminations had occurred.

## FDA

The FDA assessed the BPA literature in 2008 (FDA 2008), stating in their assessment summary that

Based on our ongoing review, we believe there is a large body of evidence that indicates that FDA-regulated products containing BPA currently on the market are safe and the exposure levels to BPA from food contact materials, including for infants and children, are below those that may cause health effects.

The FDA (2008) largely avoided the issue of current human exposure levels, giving very little attention to either the available (> 40) biomonitoring studies or the toxicokinetic studies.The FDA (2008) summarized that

There are several publications detailing measurements in biological fluid for BPA. Although [the] FDA is aware of these data and considers them extremely useful, [the] FDA also understands the experimental limitations that have been identified with regard to these data.... [The] FDA’s updated safety assessment is focused on a subpopulation, infants. Accordingly, the currently available data, which consider exposure to adults or young children (6 years of age or older), were not used or relied upon in FDA’s safety assessment.

Thus, to make their decision, the FDA included no biomonitoring studies, even those from adults that clearly indicate internal exposures to unconjugated BPA (Vandenberg et al. 2010).

## Biomonitoring Studies Should Be Used to Generate Risk Assessments

In our opinion, it is time to reassess how regulatory agencies such as the EFSA make decisions. Agencies should consider all available data in making risk assessments. As previously argued by Myers et al. (2009), the value of the peer-reviewed literature should not be judged on its ability to meet stringent regulatory criteria but on the strength of the integrated data. The large database of human biomonitoring data should be used to define human exposure levels and develop models for risk assessment. Studies in which humans were environmentally exposed to BPA are particularly relevant in this regard for assessing true human exposure levels, especially because BPA metabolism is influenced by age, sex, and physiological state (pregnant vs. nonpregnant) (Calafat et al. 2009; Vandenberg et al. 2007; Zalko et al. 2003). In addition, the two available toxicokinetic studies should be evaluated in their correct context, considering that *a*) their findings do not match findings from a large number of biomonitoring studies; *b*) there are serious inconsistencies in their methods and reported results; *c*) these studies are yet to be replicated; and *d*) these studies provide no information about fetal or neonatal exposure to BPA.

In summary, there is still significant controversy surrounding current human exposures to BPA. We propose that this controversy is not due to the lack of valid scientific biomonitoring studies, but instead stems from risk assessments generated using the same literature but applying different selection criteria that are not scientifically valid. We hope that the BPA saga will stimulate regulatory agencies to reassess how they determine the usefulness of the peer-reviewed literature and lead to the use of one integrated database of scientific information, including biomonitoring studies, to protect human health.
